# Prognostic implications of HER2NEU‐low in metastatic breast cancer

**DOI:** 10.1002/cam4.6979

**Published:** 2024-02-04

**Authors:** Zachary Neubauer, Shaakir Hasan, Robert H. Press, Arpit M. Chhabra, Jana Fox, Richard Bakst, Charles B. Simone, J. Isabelle Choi

**Affiliations:** ^1^ Thomas Jefferson School of Medicine Philadelphia Pennsylvania USA; ^2^ New York Proton Center New York New York USA; ^3^ Montefiore Medical Center Department of Radiation Oncology New York New York USA; ^4^ Mount Sinai Medical Center. Department of Radiation Oncology New York New York USA

**Keywords:** HER2 expression, hormone receptor status, metastatic breast cancer, National Cancer Database, overall survival

## Abstract

**Introduction:**

We explored characteristics and clinical outcomes of HER2‐negative and HER2‐low metastatic breast cancers using real‐world data.

**Methods:**

We queried the National Cancer Database to identify MBC patients that were HER2‐low or HER2‐negative per immunohistochemical staining. A binomial regression analysis identified demographic and clinical correlates of each subtype. A Cox multivariable regression analysis (MVA) and propensity‐match analysis were performed to identify correlates of survival.

**Results:**

Excluding missing data, 24,636 MBC patients diagnosed between 2008 and 2015 were identified; 27.9% were HER2‐negative and 72.1% were HER2‐low. There were no relevant demographic differences between the groups. HER2‐low tumors were half as likely to have concomitant hormone receptor‐positive status (*p* < 0.01). The 3‐year survival rate among hormone receptor‐negative patients was 33.8% for HER2‐low and 32.2% for HER2‐negative (*p* < 0.05), and 60.9% and 55.6% in HER2‐low and HER2‐negative cases among hormone receptor‐positive patients (*p* < 0.05), respectively. HER2‐low cases were associated with better survival on MVA (HR =0.95, 95% CI 0.91–0.99) and remained superior with propensity‐matching (HR = 0.92, 95% CI 0.89–0.96). In a subset analysis isolated to hormone receptor‐positive cases, HER2‐low remained correlated with improved survival (HR = 0.93, 95% CI 0.89–0.98) with propensity‐matched MVA. Correlates of worse survival include older age as a continuous variable (HR = 1.02, 95% CI 1.02–1.02) and Black race (HR = 1.26, 95% CI 1.20–1.32) [all *p* < 0.01].

**Conclusions:**

In the largest such analysis performed to date, our study demonstrates a small but statistically significant association with improved survival for HER2‐low tumors compared to HER2‐negative tumors in MBC.

## 1 INTRODUCTION

Approximately 20%–30% of breast cancer patients test positive for human epidermal growth factor receptor 2 (HER2).^1^ The excess production of this receptor is a known driver for cancer growth, and identification of HER2 breast cancer status can have large treatment and prognostic implications. Among metastatic ER‐positive cancers nationally, 5‐year overall survival (OS) for HER2‐negative tumors is 31.9% versus 46.0% for HER2‐positive disease. Among metastatic ER‐negative cancers nationally, 5‐year OS for HER2‐negative tumors is 12.8% versus 39.5% for HER2‐positive tumors.^2^

The mainstay HER2 targeting therapy, trastuzumab, is only indicated for HER2 positive patients. Exclusion of HER2‐low came from the landmark NSABP B‐47 phase 3 trial that did not find efficacy for trastuzumab in HER2‐low cancer.^3^ There is, however, a large demographic of HER2‐low patients for which more optimal treatment approaches are needed, with between 40% and 50% of HER2‐negative patients meeting the criteria of HER2‐low.^4^ Current guidelines characterized HER2‐positivity by immunohistochemistry (IHC) staining of 3+ or 2+, with positive gene amplification in situ hybridization (ISH) test. HER2 IHC of 0+ is considered negative. A HER2 IHC scoring 1+ or 2+ with a negative ISH test corresponds to HER2‐low disease, which is interpreted as the sample being HER2‐negative, thus contraindicating trastuzumab therapy.

HER2 is a well‐known oncogenic driver in breast cancer, historically associated with a worse prognosis before the advent of HER2‐targeted therapies. The introduction of therapies like trastuzumab has significantly improved the disease prognosis for HER2‐positive breast cancer patients. However, even under standard of care, differential cure rates between HER2‐low versus HER2‐negative metastatic breast cancers were observed in phase 2 clinical trials preceding the NSABP B‐47 phase 3 trial.^5^, ^6^ With the known differences in HER2‐positive vs HER2‐negative etiology and limited information existing on HER2‐low populations, we desired to explore the difference in prognosis between HER2‐negative and HER2‐low at the population level with current standard of care treatment. Currently, there are few papers discussing differences in survival between patients with metastatic HER2‐negative and HER2‐Low tumors, especially at a population level. We herein utilized the National Cancer Database (NCDB) to explore differential outcomes in HER2‐negative and HER2‐low patient populations with metastatic breast cancer.

## 2 METHODS

### 2.1 Patient selection

This study was exempt from institutional review board supervision due to the utilization of de‐identified data provided by the NCDB, a tumor registry managed by the American Cancer Society and American College of Surgeons. The NCDB database collects data from over 1500 hospitals accredited by the Commission on Cancer and represents data on approximately 70% of United States cancer cases.^7^, ^8^ The data used in the study are derived from a de‐identified NCDB file. The American College of Surgeons and the Commission on Cancer have not verified and are not responsible for the analytic or statistical methodology employed, or the conclusions drawn from these data by the investigator. The metastatic breast cancer patient population was selected to reduce tumor staging as a confounding variable in the analysis.

**FIGURE 1:**
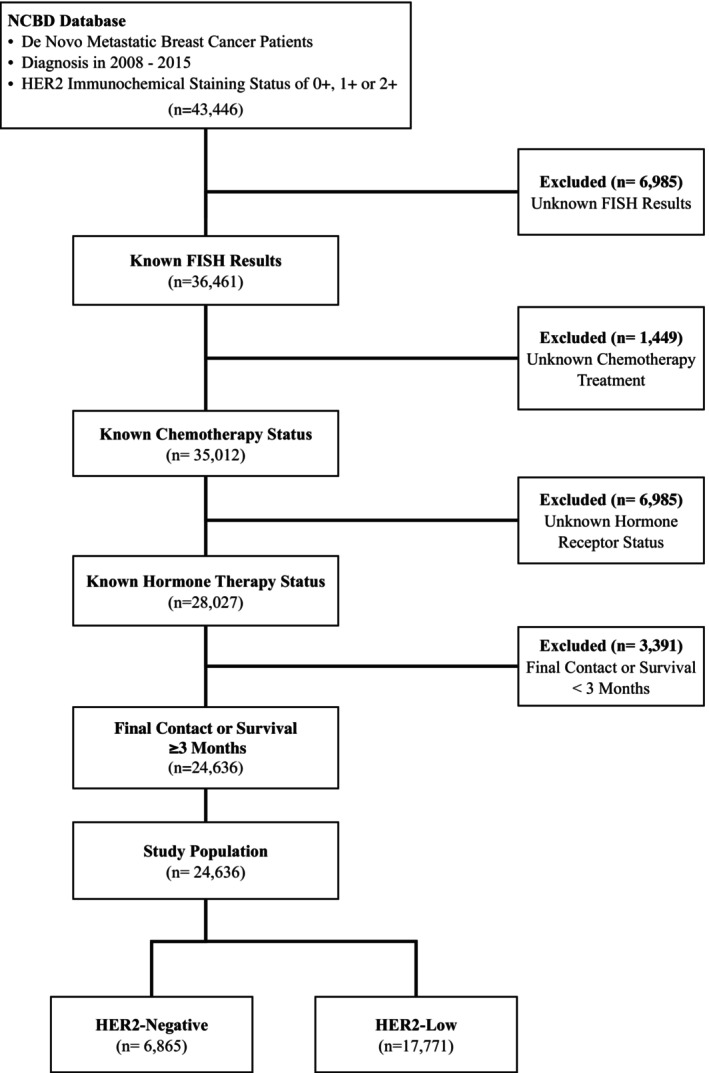
CONSORT diagram depicting the cohort selection process from the national cancer database.

We queried the NCBD database to identify metastatic breast cancer patients with confirmed HER2‐negative and HER2‐low tumors between 2008 and 2015. Inclusion criteria included known HER2 IHC status and ISH status. Patients with equivocal (2+) IHC staining and a HER2 positive FISH test are considered HER2 positive and were removed from the sample. All biopsies were from the primary tumor in de novo metastatic cases at the time of the metastatic diagnosis. Patients receiving chemotherapy or estrogen‐receptor targeting endocrine therapy were classified as systemic therapy recipients. HER2‐positive patients were excluded. Patients with unknown follow‐up were excluded. To account for immortal time bias, cases with death or last known follow‐up of less than 3 months were also excluded. A complete CONSORT diagram depicting the cohort selection process is outlined in Figure 1.

Race was defined as either White, Black, or other/unknown. Comorbidity was quantified via the Charlson/Deyo comorbidity index. All patients were metastatic and were thus categorized as stage IV as defined by the American Joint Cancer Committee 7th edition clinical staging. Population classification was based on typology published by the USDA Economic Research Service, facility type was assigned according to the Commission on Cancer accreditation category, and insurance status was reported on the admission page.

### 2.2 Statistics

Ultimately, 24,636 patients HER2‐negative and HER2‐low patients were included. Summary statistics were reported for discrete variables, and binomial regression was used to compare the two groups with respect to age, race, year treated, location, income, insurance status, Charles Deyo comorbidity scores, laterality, T stage, N stage, or use of systemic therapy. See Table 1 for population statistics for the study cohort and results of the binomial regression.

**TABLE 1:** Population Statistics and HER2 Binomial Regression for Entire Cohort.

NCDB variable	Population statistics		HER2: low vs. negative Binomial	
	N	%	Odds Ratio (95% CI)	*p* Value
HER2‐low	17,771	72.1%	_	_
ER positivity	20,161	81.8%	2.13 (1.99–2.30)	<0.001
Age at diagnosis [years]	Continuous	_	1.00 (1.00–1.01)	0.102
Charlson‐Deyo score of 0	20,423	82.9%	_	_
Charlson‐Deyo score of 1	3172	12.9%	1.03 (0.79–1.33)	0.853
Charlson‐Deyo score of 2	742	3.0%	0.88 (0.67–1.16)	0.368
Charlson‐Deyo score of 3	299	1.2%	1.03 (0.76–1.40)	0.847
Left breast	11,731	47.6%	0.99 (0.83–1.17)	0.884
Right breast	12,112	49.2%	0.97 (0.82–1.15)	0.738
Systemic therapy	20,703	84.0%	0.98 (0.90–1.07)	0.639
Year of diagnosis 2008–2011 vs 2012–2015	20,122	81.7%	1.03 (0.95–1.10)	0.509
N Stage 0	6570	26.7%	_	_
N Stage 1	7593	30.8%	0.90 (0.83–0.98)	0.016
N Stage 2	2065	8.4%	0.99 (0.90–1.08)	0.787
N Stage 3	2195	8.9%	1.01 (0.89–1.15)	0.830
N Stage unknown	6213	25.2%	1.07 (0.95–1.22)	0.271
T Stage 0	424	1.7%	_	_
T Stage 1	3231	13.1%	0.80 (0.64–1.00)	0.048
T Stage 2	5552	22.5%	1.12 (1.01–1.24)	0.029
T Stage 3	2579	10.5%	1.12 (1.03–1.23)	0.012
T Stage 4	5597	22.7%	1.13 (1.00–1.26)	0.043
T Stage unknown	7253	29.4%	1.19 (1.08–1.31)	0.001
Received chemo treatment	10,957	44.5%	0.92 (0.86–0.97)	0.002
Distance of patient residence—reporting Hospital [miles]	Continuous	_	1.00 (1.00–1.00)	0.801
Community facility	2474	10.0%	_	_
Comprehensive community facility	10,298	41.8%	0.91 (0.81–1.01)	0.078
Academic facility	7975	32.4%	0.75 (0.64–0.85)	<0.001
Integrated network facility	2607	10.6%	0.81 (0.69–0.94)	0.001
Metropolitan area	20,753	84.2%	_	_
Urban/suburban area	2773	11.3%	0.82 (0.68–0.99)	0.041
Rural area	398	1.6%	0.84 (0.68–1.03)	0.096
Female	24,291	98.6%	1.53 (1.16–2.01)	0.003
No insurance	1269	5.2%	_	_
Private insurance	10,081	40.9%	1.08 (0.84–1.39)	0.530
Medicaid	2696	10.9%	1.01 (0.81–1.26)	0.924
Medicare	9971	40.5%	1.06 (0.85–1.32)	0.605
Identify as Hispanic	2430	9.9%	1.13 (1.03–1.24)	0.013
Identify Race as White	19,265	78.2%	0.90 (0.79–1.03)	0.116
Identify Race as Black	4141	16.8%	0.97 (0.83–1.13)	0.685
Median regional household income < $38,000	4441	18.0%	_	_
Median regional household income $38,000—$47,999	5347	21.7%	1.01 (0.92–1.10)	0.883
Median regional household income $48,000—$62,999	6565	26.6%	1.05 (0.97–1.14)	0.219
Median regional household income ≤ $63,000	8156	33.1%	0.99 (0.91–1.07)	0.807
Regional high school dropout rate: ≤ 7%	5955	24.2%	_	_
Regional high school dropout rate: 7%—12.9%	7882	32.0%	1.01 (0.93–1.09)	0.894
Regional high school dropout rate: 13%—20.9%	6244	25.3%	0.94 (0.85–1.03)	0.189
Regional high school dropout rate: ≥21%	4442	18.0%	0.86 (0.77–0.97)	0.015

OS was calculated from the date of diagnosis of metastatic disease to the date of last contact or death using Kaplan–Meier curves to present the cumulative probability of survival and log‐rank statistics to assess statistical significance between HER2 status (negative versus low) groups.

The COX proportional hazards uni‐variable analysis was performed to evaluate the independent variables of patient HER2 status (negative versus low) and other covariates of interest listed in Table 1 against survival. Factors with a statistically significant correlation to survival were analyzed in the multivariable analysis. Adjusted hazard ratios (HR) and 95% confidence interval (Cl) are reported, with *α* = 0.05 used to indicate statistical significance.

The COX proportional hazards multivariable analysis was performed to evaluate the independent variables of patient HER2 status (negative versus low) and other covariates of interest found to be statistically significant in uni‐variable analysis against survival for the entire cohort.

Propensity score analysis was used to account for indication bias caused by lack of randomization, as supported by D Agostino's and Cohen's work on propensity score methods.^9^, ^10^ Propensity scores were calculated by multivariable logistic regression to provide a score reflecting the conditional probability of being HER2‐negative or HER2‐low. Subsequently, we constructed a Cox proportional hazards model adjusting for propensity score with inverse probability weighting.^11^ To avoid overcorrection, only factors significant in uni‐variable survival analysis and not included in the propensity score, were included in the propensity‐adjusted model.

An additional study of the estrogen receptor (ER)‐positive subgroup was performed. A uni‐variable COX proportional hazards analysis was performed to evaluate the independent variables of patient HER2 status (negative versus low), and the other covariates of interest listed in Table 1, against survival for the ER‐positive cohort. Variables found to be statically significant on UVA were entered into a COX proportional hazards multivariable analysis to evaluate the independent variables of patient HER2 status (negative versus low), and the other covariates, against survival for the ER‐positive cohort. See Table 3 for the complete list of variables and results for the MVA of the ER‐positive cohort.

A propensity score analysis was performed on the subgroup of patients who were hormone receptor positive. Using the same method as described for the broader cohort, propensity scores were calculated by multivariable logistic regression to provide a score reflecting the conditional probability of being HER2‐negative or HER2‐low. A pseudo‐population of ER‐positive patients with a representative distribution of confounding variables in both HER2‐low and HER2‐negative groups was created, and a Cox proportional hazards model adjusting for propensity score with inverse probability weighting was constructed. Only factors significant in uni‐variable survival analysis not included in the propensity score were included in the propensity‐adjusted model.

## 3 RESULTS

### 3.1 Patient characteristics

Twenty four thousand and six thirty six MBC patients diagnosed between 2008 and 2015 were identified, 6865 (27.9%) of whom were HER2‐negative and 17,771 (72.1%) of whom were HER2‐low. Baseline patient characteristics for all included patients are shown in Table 1; in summary, the median age was 62 years old, 24,291 (98.6%) were female, and 20,161 (81.8%) were ER‐positive.

Binomial regression was performed to analyze variable correlations between HER2‐low and HER2‐negative status. The analysis found no significant differences between the two groups with respect to age, race, year treated, location, income, insurance status, Charles Deyo comorbidity score, laterality, or use of systemic therapy. Binomial regression analysis found HER2‐low patients were more likely to be Hispanic (OR = 1.13, 95% CI 1.03–1.24) than HER2‐negative patients. N‐stage 1 was less likely in HER2‐low tumors (OR = 0.90 95% CI 0.83–0.98), but there was no correlation in other N‐stages. T‐stage 1 was less likely in HER2‐low tumors (OR = 0.80 95% CI 0.64–1.00), whereas all other T Stages correlated positively with HER‐low status (HR = 1.12 to 1.19). HER2‐low status was more likely to have concomitant hormone receptor‐positive status (HR = 2.13, 95% CI 1.99–2.30). Table 1 depicts the population statistics and the binomial regression analysis results for the cohort.

### 3.2 Survival—full cohort

A Kaplan–Meier function was used to determine survivability within the patient group.

**FIGURE 2:**
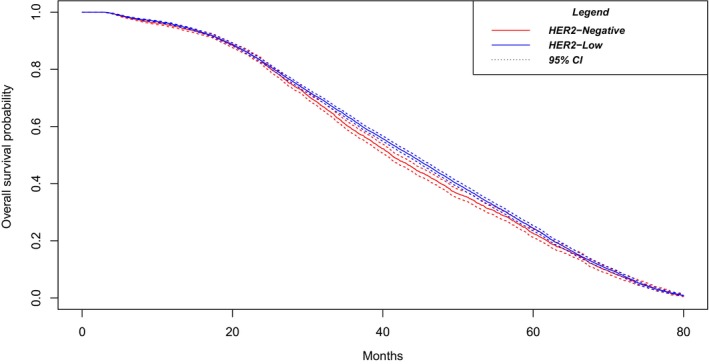
Kaplan Meyer Survival for the HER2‐Negative versus HER2‐Low metastatic breast cancer patients. Median survival was 41.17 (95% CI 40.34–42.12) months for HER2‐negative vs 43.7 (95% CI 43.0–44.2) months for HER2‐low patients, *p*‐value of 0.009.

For the entire cohort, median survival was 43.01 (95% CI 42.41–43.53) months. For the HER2‐negative patients, median survival was 41.17 (95% CI 40.34–42.12) months. Comparison of survival between HER2‐low and HER‐2 negative groups resulted in a p‐value of 0.009. For the HER2‐low patients, median survival was 43.7 (95% CI 43.0–44.2) months. The 3‐year survival rate among hormone receptor‐negative patients was 33.8% for HER2‐low and 32.2% for HER2‐negative (*p* < 0.05), and 60.9% and 55.6% in HER2‐low and HER2‐negative cases among hormone receptor‐positive patients (***p*** < 0.05), respectively. Figure 2 depicts Kaplan–Meier survival curves for HER2‐negative and HER2‐positive patient populations.

Uni‐variable analysis showed HER2‐low patients had significantly better survival (HR = 0.88, 95% CI 0.85–0.92) than HER2‐negative patients. ER‐positivity had the largest survival benefit (HR = 0.46, CI 95% 0.44–0.48) of any variable examined.

HER2‐low status and 7 other variables were included in the multivariable analysis. The analysis found the largest significant correlations of increased survival continued being ER positivity (HR = 0.48 95% CI 0.46–0.51) and with HER2‐low status (HR = 0.95, 95% CI 0.91–0.99). Patients who identified their race as Black had lower survival (HR = 1.26, CI 95% 1.20–1.32) than patients who identified as White. Older age correlated with worse survival as a continuous variable (HR = 1.02, 95% CI 1.02–1.02), Refer to Table 2 for the complete list of factors in the MVA.

**TABLE 2:** Survival MVA results for entire cohort.

	Multi variable analysis	
NCDB variable	Hazard Ratio (95% CI)	*p* Value
HER2‐low	0.95 (0.91–0.99)	0.0082
Age at diagnosis [years]	1.02 (1.02–1.02)	<0.001
ER Positive	0.48 (0.46–0.51)	<0.001
Charlson‐Deyo score of 1	1.30 (1.24–1.37)	<0.001
Charlson‐Deyo score of 2	1.62 (1.48–1.78)	<0.001
Charlson‐Deyo score of 3	1.84 (1.59–2.11)	<0.001
Received chemo treatment	1.19 (1.14–1.25)	<0.001
Systemic therapy	1.26 (1.18–1.33)	<0.001
Year of diagnosis 2008–2011 vs 2012–2015	1.10 (1.05–1.15)	<0.001
Identify Race as Black	1.26 (1.20–1.32)	<0.001

HER2 status propensity scored multivariable analysis showed HER2‐low status continued to be associated with increased survival (HR = 0.92, 95% CI 0.89–0.96).

### 3.3 Survival—ER‐positive cohort

A separate analysis was conducted on the subset of the cohort who are ER‐positive.

Uni‐variable analysis of the ER‐positive cohort found HER2‐low status continued to be significantly correlated with improved survival (HR = 0.94, 95% CI 0.90–0.98). Patients who identified their race as black had non‐statistically significantly decreased survival (HR = 1.04 CI 95% 0.99–1.10).

Multivariable analysis of the ER‐positive cohort found statistically significant increased survival with HER2‐low status (HR = 0.92, 95% CI 0.87–0.96) over HER2‐negative. Patients who identified as Hispanic were found to have significantly improved survival (HR = 0.90 CI 95% 0.83–0.97). Refer to Table 3 for the MVA results in the ER‐positive subgroup.

**TABLE 3:** MVA results for estrogen receptor positive cohort.

	Multi variable analysis	
NCDB variable	Hazard Ratio (95% CI)	*p* Value
HER2‐low	0.92 (0.87–0.96)	0.001
Age at diagnosis [years]	1.02 (1.02–1.02)	<0.001
Identify as Hispanic	0.90 (0.83–0.97)	0.005
Identify race as Black	0.81 (0.73–0.91)	<0.001
N Stage 1	1.22 (1.15–1.29)	<0.001
N Stage 2	1.13 (1.03–1.22)	0.007
N Stage 3	1.23 (1.13–1.33)	<0.001
T Stage 0	_	_
T Stage 1	1.65 (1.36–2.00)	<0.001
T Stage 2	1.87 (1.54–2.26)	<0.001
T Stage 3	2.10 (1.73–2.55)	<0.001
T Stage 4	2.15 (1.78–2.59)	<0.001
Charlson‐Deyo score of 0	_	_
Charlson‐Deyo score of 1	1.28 (1.21–1.36)	<0.001
Charlson‐Deyo score of 2	1.50 (1.35–1.67)	<0.001
Charlson‐Deyo score of 3	1.66 (1.42–1.95)	<0.001
Systemic Therapy	0.97 (0.91–1.05)	0.45
Year of Diagnosis 2008–2011 vs 2012–2015	1.06 (1.01–1.12)	0.025
No Insurance	_	_
Private insurance	0.72 (0.65–0.79)	<0.001
Medicaid	0.84 (0.76–0.92)	<0.001
Medicare	0.75 (0.62–0.92)	0.005
Distance of patient residence—reporting Hospital [miles]	1.00 (1.00–1.00)	0.866
Urban/suburban area	0.99 (0.92–1.06)	0.757
Rural area	1.05 (0.90–1.24)	0.524

Multivariable analysis of the HER2 status propensity scored, ER‐positive population showed HER2‐low status continued to be associated with increased survival (HR = 0.93, 95% CI 0.89–0.98) in the ER‐positive subpopulation.

## 4 DISCUSSION

Since its introduction, trastuzumab, the mainstay HER2 targeting therapy, was only indicated for HER2‐positive patients. Of note, the NSABP B‐47 phase 3 trial did not find efficacy with trastuzumab added to treatment in HER2‐low cancer patients.^3^

However, in August 2022, Fam‐trastuzumab deruxtecan was approved by the FDA for patients with unresectable or metastatic HER2‐low breast cancer who have received prior chemotherapy in the metastatic setting or developed disease recurrence. In the randomized DESTINY‐Breast04 trial, 557 HER2‐low patients with metastatic breast cancer who had received chemotherapy were enrolled. The trial found the antibody‐drug conjugate fam‐trastuzumab deruxtecan led to improved OS compared to treatment with physician's choice of chemotherapy. Among those receiving fam‐trastuzumab deruxtecan and chemotherapy, the median OS was 23.4 months versus 16.8 months, respectively. Furthermore, the median progression‐free survival was 9.9 months in those receiving fam‐trastuzumab deruxtecan versus 5.1 months for chemotherapy. The benefit of fam‐trastuzumab deruxtecan over chemotherapy was noted in both hormone receptor‐positive and hormone receptor‐negative subgroups, with 11% of the study participants having had hormone receptor negative tumors. The inclusion of Fam‐trastuzumab deruxtecan for the aforementioned HER2‐low patients was endorsed in new guidelines from the American Society of Clinical Oncology.^12^, ^13^, ^14^

With HER2 being a known target for treatment resulting in superior outcomes, and with the recent indication of Fam‐trastuzumab deruxtecan for specific HER2‐low cancers, we desired to explore the difference in prognosis between HER2‐negative and HER2‐low at the population level with current standard of care treatment. Our analysis of patients in the National Cancer Database (NCDB) shows patients with HER2‐low who received the current standard of care treatment between 2008 and 2015 had greater survival than HER2‐negative patients in multivariable analysis. The difference in prognosis suggests significant differences in the etiology and biology of HER2‐Low and HER2‐negative metastatic breast cancer that should be explored for future treatment exploitation.

### 4.1 Population

Binomial distribution found a correlation between HER2‐low status and Hispanic identification (OR = 1.13, 95% CI 1.03–1.24). This finding adds to other analyses that have found an association between Hispanic identification and HER2 status. A 2022 study of 33,976 women with metastatic breast cancer found that Hispanic women were more likely to be HER2‐negative than HER2‐positive (31% vs. 28%, *p* < 0.01); this study did not differentiate between HER2‐low and fully HER2‐negative cancers.^15^

Hormone receptor‐positive cancers are known to have improved survival among metastatic breast cancer patients.^2^ Our binomial analysis found patients with ER‐positive tumors were more likely to be HER2‐low (OR = 2.13 95% CI 1.99–2.30). This result is consistent with previous work on nonmetastatic tumors, which found a higher prevalence of progesterone receptor expression in HER2‐low than in HER2‐negative tumors.^16^, ^17^

### 4.2 Survival

Our analysis found HER2‐low status was significantly correlated with increased survival in uni‐variable analysis, multivariable analysis, and propensity matched multivariable analysis, providing strong evidence for the legitimacy of HER2‐low status as a prognostic indicator. Our finding of HER2‐low status having greater survival than HER2‐negative status is consistent with previous studies. A 2022 retrospective analysis of 391 breast cancer patients concluded HER2‐low cancers correlated with lower‐grade cancers and longer OS than HER2‐negative patients.^17^ Similarly, a 2022 study analyzing metastatic breast cancer patients in the NCDB database found HER2‐negative status was associated with higher survival in both hormone receptor negative and positive groups.^18^

A 2021 analysis of the PRAEGNANT prospective registry of advanced breast cancers found no significant difference in survival between HER2‐low vs HER2‐negative advanced triple‐negative breast cancers or in advanced hormone receptor–positive breast cancers.^19^ We suspect this discrepancy may arise from the larger power of our study (*n* = 2033 vs. our 24,636).

Our analysis found patient race was a significant variable impacting survival. In the multivariable analysis, persons identifying as Black (HR = 1.26, 95% CI 1.20–1.32) had worse survival than persons who identified their race as White. There was no correlation between HER2‐low status and patient identifying as Black (OR = 0.97, 95% CI 0.83–1.13). This is consistent with previous research, with one 2022 study of 33,976 metastatic breast cancer patients finding Black women had the greatest (89%) five‐year cumulative incidence of cancer‐specific death compared to women of other racial identifications.^15^ However, a 2015 multivariable analysis of risk factors among 4364 patients with metastatic breast cancer found patients identifying as Black had poorer survival for HER2‐positive but not HER2‐negative tumors. This discrepancy may be due to differences in our sample size, or the greater number of socioeconomic factors, like rural–urban location and median income, included in their analysis.^20^

### 4.3 Limitations

While this report is the largest such analysis performed to date on this topic among metastatic breast cancer patients, several limitations exist: Our analysis relied heavily on the categorization of patients between HER2‐low and HER2‐negative tumors. A multi‐center, retrospective study in 2022 assessed 233 tumor samples with historic HER2‐low and HER2‐negative scoring that were rescored using IHC and nonstaining assays and showed an 82.3% concordance between historic and regraded samples.^21^ This categorization discrepancy is a limitation on the accuracy of our analysis. Development of new assays may be needed to increase the precision of HER2 status classification in the future. “Metastatic breast cancer” disease does not represent a prognostic monolith, and factors like mutation etiology, and the number or location of metastatic sites were not investigated.

Our conclusions are generated from large datasets with statistical significance but are subject to several limitations, including potential selection bias, as well as the lack of several factors in the NCDB dataset that may alter the interpretation of our results, including initial treatment response, salvage therapies, and disease recurrence. For example, the current data cannot account for prophylactic treatment, which may be a confounding variable.

Our study used multivariable analysis in an effort to isolate the impact of HER2 status from the influence of statistically significant risk factors such as age. Additionally, analysis with propensity score matching reduces the likelihood of confounding variables or bias changing the study result, thus providing further evidence for the significance of HER2‐low vs HER2‐negative status being a significant variable for prognosis. Furthermore, we employed rigorous and commonly employed statistical methods and detailed our approach with supporting literature. However, we acknowledge our statistical methods, while ubiquitous in medical research, are controversial in contemporary statistical literature as no single multivariable analysis methodology has gained consensus support. Multivariable analysis or propensity matching analysis cannot substitute for the randomness central to accounting for confounding variables in large clinical trials, and we encourage future randomized clinical trials on this topic.

## 5 CONCLUSION

To our knowledge, this paper is the largest analysis to date describing patient characteristics and prognosis associated with HER2‐negative and HER2‐low MBC. Consistent with recent data in non‐MBC,^22^ our study demonstrates a small but statistically significant association with improved survival for HER2‐low tumors compared to HER2‐negative tumors. This difference in outcomes highlights the possibility for an etiological and biological distinction between HER2‐negative and HER‐low.

There is a large demographic of HER2‐low patients, with 40–50% of HER2‐negative patients meeting the criteria of HER2‐low.^4^ Differentiating between HER2‐negative and HER2‐low populations may open doors for novel treatments or management strategies that could improve outcomes in this large HER2‐low patient population. This posit is coming to reality with the 2022 FDA approval of Enhertu (fam‐trastuzumab‐deruxtecan‐nxki), the first treatment for unresectable or metastatic HER2‐low breast cancer.^14^ Further developments specific to HER2‐low are likely.

## AUTHOR CONTRIBUTIONS

**Zachary Neubauer:** Data curation (lead); formal analysis (lead); methodology (supporting); project administration (lead); writing – original draft (lead). **Shaakir Hasan:** Conceptualization (lead); data curation (supporting); formal analysis (supporting); methodology (lead); writing – original draft (supporting); writing – review and editing (equal). **Robert H. Press:** Writing – review and editing (supporting). **Arpit Chhabra:** Writing – review and editing (supporting). **Jana Fox:** Writing – review and editing (supporting). **Richard Bakst:** Writing – review and editing (supporting). **Charles B. Simone:** Writing – review and editing (equal). **J. Isabelle Choi:** Writing – review and editing (supporting).

## FUNDING INFORMATION

The production of this paper did not involve funding from any organization.

## CONFLICT OF INTEREST STATEMENT

All authors attest to having no conflicts of interest in the publication of this paper.

## CONSENT

No clinical trials or identifiable patient information was used in the production of this paper.

## Supporting information


Data S1.

Table S1.

## Data Availability

The data source and methods were evaluated and determined to be exempt from Internal Review Board approval at associated institutions.
