# Supplementary material for: Prognostic implications of HER2NEU‐low in metastatic breast cancer
Source: Cancer Med. 2024 Feb 4;13(2):e6979. doi: 10.1002/cam4.6979 (PMC10839127; doi:10.1002/cam4.6979)
Supplement: Supplementary file 1 — Data S1. [file CAM4-13-e6979-s002.docx]

# Supplements


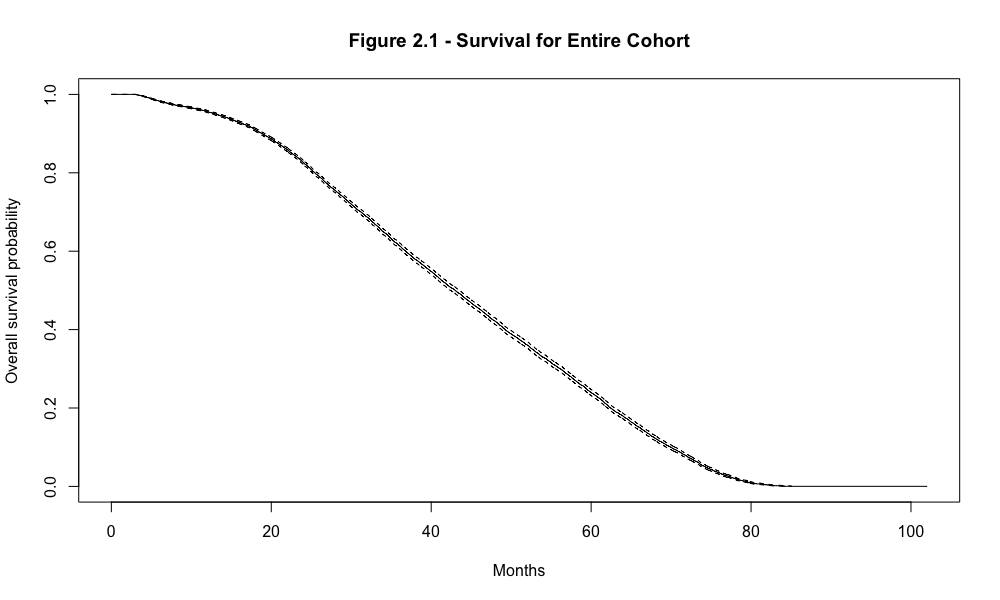


Supplement Figure: Kaplan Myer Curve For Entire Cohort. Median survival was 43.01 (95% CI 42.41 - 43.53) months
